# MSH2 Overexpression Due to an Unclassified Variant in 3’-Untranslated Region in a Patient with Colon Cancer

**DOI:** 10.3390/biomedicines8060167

**Published:** 2020-06-19

**Authors:** Raffaella Liccardo, Antonio Nolano, Matilde Lambiase, Carlo Della Ragione, Marina De Rosa, Paola Izzo, Francesca Duraturo

**Affiliations:** 1 Department of Molecular Medicine and Medical Biotechnology, University Federico II, via Pansini 5, 80131 Naples, Italy; raffaella.liccardo@unina.it (R.L.); nolano@ceinge.unina.it (A.N.); lambiase@ceinge.unina.it (M.L.); marina.derosa@unina.it (M.D.R.); paola.izzo@unina.it (P.I.); 2 CEINGE-BiotecnologieAvanzate, via G. Salvatore 486, 80145 Naples, Italy; 3 UOC Pathological Anatomy, Azienda Ospedaliera di Rilievo Nazionale (AORN) “A. Cardarelli”, via A. Cardarelli 9, 80131 Naples, Italy; c.dellaragione@hotmail.it

**Keywords:** MSH2 3’UTR variant, hereditary colon cancer, Lynch syndrome, MSH2 protein, over expression MSH2, MMR gene, MMR complex deficiency, MSH2 unclassified variants

## Abstract

Background: The loss or low expression of DNA mismatch repair (MMR) genes can result in genomic instability and tumorigenesis. One such gene, MSH2, is mutated or rearranged in Lynch syndrome (LS), which is characterized by a high risk of tumor development, including colorectal cancer. However, many variants identified in this gene are often defined as variants of uncertain significance (VUS). In this study, we selected a variant in the 3′ untranslated region (UTR) of MSH2 (c*226A > G), identified in three affected members of a LS family and already reported in the literature as a VUS. Methods: The effect of this variant on the activity of the MMR complex was examined using a set of functional assays to evaluate MSH2 expression. Results: We found MSH2 was overexpressed compared to healthy controls, as determined by RTqPCR and Western blot analyses of total RNA and proteins, respectively, extracted from peripheral blood samples. These results were confirmed by luciferase reporter gene assays. Conclusions: We therefore speculated that, in addition to canonical inactivation via a gene mutation, MMR activity may also be modulated by changes in MMR gene expression.

## 1. Introduction

The loss or low expression of MSH2 in particular, but also that of other DNA mismatch repair (MMR) genes, results in genomic instability and a predisposition to cancer [1,2]. It has also been described that the overexpression of *MLH1* and/or *MSH2* induces apoptosis and/or a mutator phenotype with genetic instability [3,4,5]. At the somatic level, genomic instability is evident, especially in repeated DNA sequences, known as microsatellite sequences. Indeed, microsatellite instability (MSI) is an important molecular marker for the characterization of a mutator phenotype linked to *MMR* genes [6]. A defective MMR system mainly results from mutations in the same *MMR* genes and is the basis of Lynch syndrome (LS). LS is an autosomal dominant condition caused by a defect in one of the *MMR* genes and is characterized by a high lifetime risk of tumor development, especially colorectal cancer (20–70%), endometrial cancer (15–70%), and other extracolonic tumors (15%) [7]. The molecular characterization of LS patients relies on the identification of point mutations and large rearrangements in the coding regions of the *MMR* genes, *MLH1*, *MSH2*, *PMS2,* and *MSH6* [8,9,10,11,12,13]. 

The defects are often caused by mutations in the coding regions of *MMR* genes or by the promoter methylation of these genes. However, in many cases, despite the presence of a hypermutable phenotype in a patient, no mutations/hypermethylation of *MMR* genes can be detected [14,15]. 

It is noteworthy that, in addition to canonical inactivation via gene mutation, MMR activity can also be modulated by changes in *MMR* gene expression [16]. To date, many hypotheses on other causes that determine loss of function in the MMR system have been postulated. Variants in some genetic regions, such as in the 3’ untranslated region (UTR), may impair the binding of putative transcriptional factors or micro (mi)RNA involved in the regulation of gene expression. In this regard, it very interesting to note a study that demonstrated a regulatory mechanism existing between miR- 422a and the *MLH1* gene, following the identification of a variant of uncertain significance (VUS) in the 3’ UTR of the *MLH1* gene in a LS patient [17]. Therefore, the functional study of VUS in the 3’UTR of *MMR* genes may allow us to understand the pathogenetic significance of these variants. Here, we report a functional study of a variant identified in the 3′ UTR of *MSH2* (c*226A > G), already described as a variant of uncertain significance in an international database of *MMR* variants (*www.insight-database/varints.org*). This variant was found in three patients of the same family with a LS-related cancer. 

## 2. Materials and Methods 

The Clinical Department of Laboratory Medicine of the hospital affiliated to Federico II University (Naples, Italy) recruited the subjects after receiving authorization from the local ethics committee “Comitato etico per le attività Biomediche Carlo Romano” of the University of Naples, Federico II (protocol no. 120/10, approval November 2010). Once the authorization was obtained, the study received ethical approval, the participants were informed, and written consent was obtained.

The experiments were performed on DNA and on cDNA extracted from peripheral blood lymphocytes. 

### 2.1. DNA Extraction from Patient Samples

Total genomic DNA was extracted from 4 mL peripheral blood lymphocytes collected in EDTA using a BACC2 Nucleon Kit (Amersham Life Science, Buckinghamshire, UK), according to the manufacturer’s recommendations, and from formalin-fixed and paraffin-embedded (FFPE) tumor tissues using standard methods. The DNA was precipitated with two volumes of absolute ethanol and the pellet was washed with ethanol at 70% and resuspended in sterilized TE buffer (Tris 10 mM pH7.5-EDTA 1 mM pH8) [9].

### 2.2. DNA Amplification and Microsatellite Analysis

MSI was tested on paired samples of lymphocyte DNA and in paraffin-embedded tumor tissues of the colon. The MSI status was evaluated with a fluorescent multiplex system comprising five mononucleotide repeats (BAT-25, BAT-26, NR-21, NR-24, Bat40, and TGFβRII), four dinucleotide repeats (D2S123, D18S58, D5S346, and D17S250) and two tetranucleotide repeats (TPOX and TH01) using the CC-MSI kit (AB ANALITICA, Padova, Italy), and subsequent capillary electrophoresis analysis using an ABI 3130 Prism (Applied Biosystems, Fisher Thermo Scientific, Waltham, MA, USA). Tumors were classified as “highly unstable” (MSI-H), if at least 30% of the markers showed instabilities and “with low levels of instability” (MSI-L), if at least 10% of the markers showed instabilities. If no allele difference between the DNA extracted from normal and tumorous tissues was observed, tumors were classified as microsatellite stable (MSS) [9].

### 2.3. Immunohistochemistry

IHC analysis was performed on a Benchmark XT automatized immunostainer (Ventana Medical Biosystems, Tucson, USA). The primary antibody used was anti-MSH2 mouse monoclonal clone G219–1129. This antibody is supplied by the manufacturer optimally pre-diluted (3.04 µg/mL) to be compatible with VENTANA detection kits (Sigma-Aldrich). The detection system used was an iVIEW DAB Detection Kit (Ventana) based on the streptavidin–biotin-conjugated system. The antigen–positive complexes were detected by the addition of the DAB chromogen (diaminobenzidine) and its substrate (H_2_O_2_). The samples were finally counterstained with hematoxylin. Nuclear staining was observed using an optical microscope with positivity represented by the presence of brown staining [9]. This positivity was compared with blue nuclear epitopes in which the specific antigen was not present. The internal positive control was represented by lymphocytes, stroma, and functional mucosal crypts, while the negative control was obtained by slides without primary antibody. Strong, moderate, weak, or negative staining is revealed by this method.

### 2.4. RNA and Protein Analysis

Total RNA was extracted from 4 mL peripheral blood lymphocytes of the patients and from three normal controls using QIAzol reagent, according to the manufacturer’s instructions (Qiagen, Hilden, Germany). cDNA was synthesized using 1 µg total RNA, 500 ng random hexamers, and 1 µL SuperScript III reverse transcriptase (Invitrogen Life Technologies, Carlsbad, CA, USA), in the presence of 4 µL 5× RT buffer, 1 µL dithiothreitol (0.1 M), and 1 mM deoxynucleotide triphosphates (Invitrogen Life Technologies, Inc.). The reaction was run on a PCR thermocycler for 50 min at 42 °C in a 20 µL reaction volume, heated to 70 °C for 15 min, and subsequently chilled on ice. Quantitative RNA analysis was performed by RT-PCR on a CFX96 Real-Time System (Bio-Rad-Laboratories, Inc., Hercules, CA, USA). Two forward and reverse primers for *MSH2* cDNA quantification were carried out by amplifying fragments spanning exons 4–5 and 13–14 (primer pair 4F-ACCGGTTGTTGAAAGGCAAA and 5R-TTGATTACCGCAGACAGTGATG; 13F-TGGTGACAGTCAATTGAAAGGA and 14R-CCCATGCTAACCCAAATCCA). A calibration curve to assess the efficiency of the PCR reaction was performed on at least three serial dilutions (1:10) of the reverse-transcriptase products. All primer sets had efficiencies of 100% ± 10%. Each RT-PCR was performed in triplicate in a 20 µL reaction mix containing 12.5 µL of 2× SYBR Green I PCR Master mix (Bio-Rad Laboratories, Inc.), 0.38 µL of a 20 µM primer mix, 2 µL of cDNA (5 ng/µL), and 7.12 µL of nuclease-free water. The cycling conditions consisted of an initial denaturation step at 95 °C for 3 min, followed by 40 cycles (95 °C for 15 s, 62 °C for 30 s, and 82 °C for 20 s) and 80 cycles performed according to the standard protocols for melting curve analysis. The CT values were determined by automated threshold analysis and the data were analyzed with the CFX Manager software version 2.1 (Bio-Rad Laboratories, Inc.). The relative expression of the target transcript was calculated with the comparative Ct method using a cDNA fragment from the glucuronidase (*GUS*) housekeeping gene as a control. The specificity of qPCR products was evaluated by melting curve analysis [9]. 

Total protein extracts (nuclear and cytosolic proteins) were obtained from the same blood sample used for the RNA isolation of affected patients, using QIAzol reagent (Qiagen) according to the manufacturer’s instructions. The evaluation of protein concentration was performed by spectrophotometer analysis at 595 nm according to the Bradford method using the Bio-Rad Protein Assay Reagent (Bio-Rad Laboratories, Inc.). A total of 50 µg of proteins were separated by SDS-polyacrylamide gel electrophoresis and blots were prepared on an Amersham Hybond-ECL nitrocellulose membrane (Amersham Pharmacia Biotech, Inc./GE Healthcare Bio-Sciences Corp., Piscataway, NJ, USA) [18]. Membranes were incubated overnight at 4 °C with primary antibody against MSH2 (1:100, mouse monoclonal anti-human, clone GB12; Calbiochem, EMD Chemicals, Inc., Merck KGaA, Darmstadt, Germany) and subsequently normalized with anti-actin antibody (1:1000, rabbit polyclonal anti-human; clone sc-1615; Santa Cruz Biotechnology, Inc., USA). The antigen–antibody complexes were visualized with the ECL-Immobilon chemiluminescence reagents (Millipore, Life Science of Merck KGaA, Darmstadt, Germany) and subsequent autoradiography. Western blotting bands were quantified by ImageJ software.

### 2.5. Luciferase Constructs and Reporter Assay

The *MSH2* 3′ UTR of one of the three heterozygous carriers of the mutation, c.*226 A > G, was amplified by PCR with a primer pair containing *XhoI* and *NotI* restriction sites. Oligonucleotide sequences were as follows: *XhoI*-3′ UTR *MSH2* forward: ATACTCGAGAAAATCCCAGTAATGGAATG and *NotI*-3′ UTR *MSH2* reverse: ATAGCGGCCGCTTCAAATTCCACAAACTACA. The PCR product was cloned into the PSICHECK2 vector (Promega, Madison, WI, USA) downstream of the *Renilla* luciferase coding region (hRluc). The orientation of the wild type (WT) and mutated (MUT) inserted products was established by digestion and confirmed by sequencing. The PSICHECK2 constructs with additional mutations in the *MSH2* 3′ UTR region were generated using the QuiKChange Mutagenesis kit (Agilent Technologies, Santa Clara, CA, USA). Oligonucleotide sequences for site-directed mutagenesis were as follows: 3′UTR *MSH2* MUT1 forward, GGACTGTTTGCAATTGACATAGGTACTgATAAGTGATGTGCTG and reverse, CAGCACATCACTTATcAGTACCTATGTCAATTGCAAACAGTCC; 3′UTR *MSH2* MUT2 forward, GGACTGTTTGCAATTGACATAGGTCCGgATAAGTGATGTGCTG, and reverse, CAGCACATCACTTATcCGGACCTATGTCAATTGCAAACAGTCC (patient mutated base is in lowercase, and additional mutated bases are in bold). Luciferase activity was measured at 48 h after transfection using a dual luciferase reporter assay (Promega) according to the manufacturer’s instructions and performed on a 20n/20n luminometer (Turner BioSystems, Sunnyvale, CA, USA). Relative luciferase activity was calculated by normalizing the *Renilla* luminescence to the firefly luminescence [19]. 

### 2.6. Statistical Analysis

Data from real-time quantitative PCR, Western blot analysis, and luciferase assays were analyzed using GraphPad Prism 6.0 software (GraphPad Software, Inc.). The mean values (± S.D.) were calculated. Statistical significance was determined using Student’s t test or one-way analysis of variance (ANOVA). Data were considered statistically significant when p-values were ≤ 0.05

## 3. Results

### 3.1. Selected Patients

Three patients of a LS family were selected for this study. These patients were carriers of the variant c*226A > G in the 3′ UTR of *MSH2*. All patients were negative for pathogenic point mutations in the *MMR* genes (*MSH2*, *MSH6*, *MLH1* and *PMS2*) and no large rearrangements in these genes were identified. 

**Figure 1:**
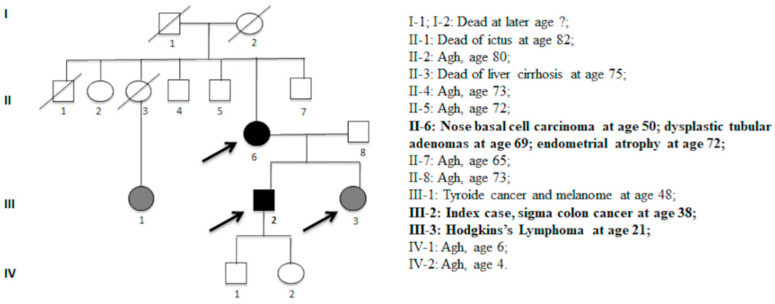
Pedigree with the segregation of the c.*226A > G variant. Symbols and abbreviations used are denoted as fellow: arrows, analysed members of family; black symbol, colorectal cancer or cancer associated with HNPCC; gray symbols, adenomas or cancer not associated with HNPCC. Numbers next to symbols denote age at onset; the phenotype patients are reported next to pedigree. Agh, apparent good health.

The index case of the family was a man who had developed a sigma degenerated polyp with severe dysplasia and in situ adenocarcinoma at the age of 38 years. The mother developed a nose basal cell carcinoma at the age of 50 years, two colon mildly dysplastic tubular adenomas at the age of 69 years, and endometrial atrophy at the age of 72 years. The sister developed Hodgkin lymphoma at the age of 21 years, as shown in Figure 1. The three control subjects were used in this experimental study. The patients and the controls were of Caucasian origin and were from the Campania geographic region (Southern Italy).

In our laboratory, this variant has also been identified in another patient who developed a grade 3 (G3) endometrial cancer at 49 years of age, and who also had a familial cancer history.

### 3.2. Microsatellite and Immunohistochemistry Analysis

**Figure 2:**
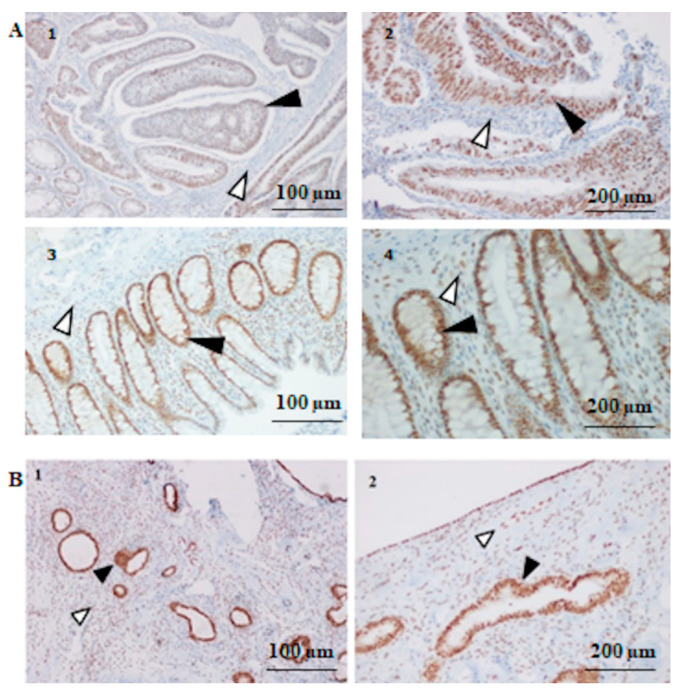
MSH2 immunohistochemistry (IHC) analysis. (**A**) MSH2 IHC results in index patient III-2. (1) Moderate positive IHC in the colon tumor cells (filled arrow heads point) 100 μm and (2) 200 μm; (3) strong positive IHC in the normal mucosa cells (filled arrow head point) of the patient 100 μm and (4) 200 μm, compared with IHC+ internal stromal cells (open arrow head point). (**B**) MSH2 IHC results in patient II-6. (1) Strong positive staining in the endometrial atrophic polyp cells (filled arrow head point) 100 μm and (2) 200 μm, compared with IHC+ internal stromal cells (open arrow head point).

The Microsatellite analysis (MSI) was performed on two patients, the index case and his mother, respectively. The MSI-Low status was found, two dinucleotide repeats (D5S346 and D18S58) were unstable in both patients, on the sigma tissue of the index case and on the endometrial tissue of his mother. Moreover, the immunohistochemistry analysis (IHC) of the paraffin-embedded tissue stions of the two above patients revealed a strong intensity of MSH2 staining, as shown in Figure 2. 

### 3.3. Overexpression of MSH2 Gene and Overexpression of MSH2 Protein

**Figure 3:**
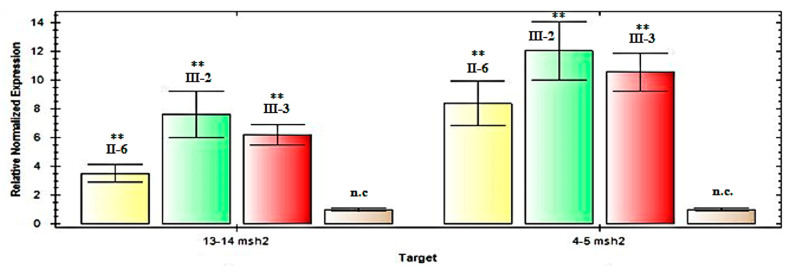
q-Real-Time PCR analysis of the *MSH2* mRNA in the patients with the c.*226A > G variant. Relative expression, calculated using the comparative Ct method, of *MSH2* cDNA, including fragments 13–14 and 4–5, normalized to β-glucuronidase levels, in patients II-6, II-2, III-3 and in an average of three normal controls (n.c.). The results represent the average of three independent experiments ± standard deviation. Patient numbering corresponds to that adopted in the pedigree, as shown in Figure 1. ** Statistical significance was determined by one-way ANOVA (*p*-values were < 0.05).

**Figure 4:**
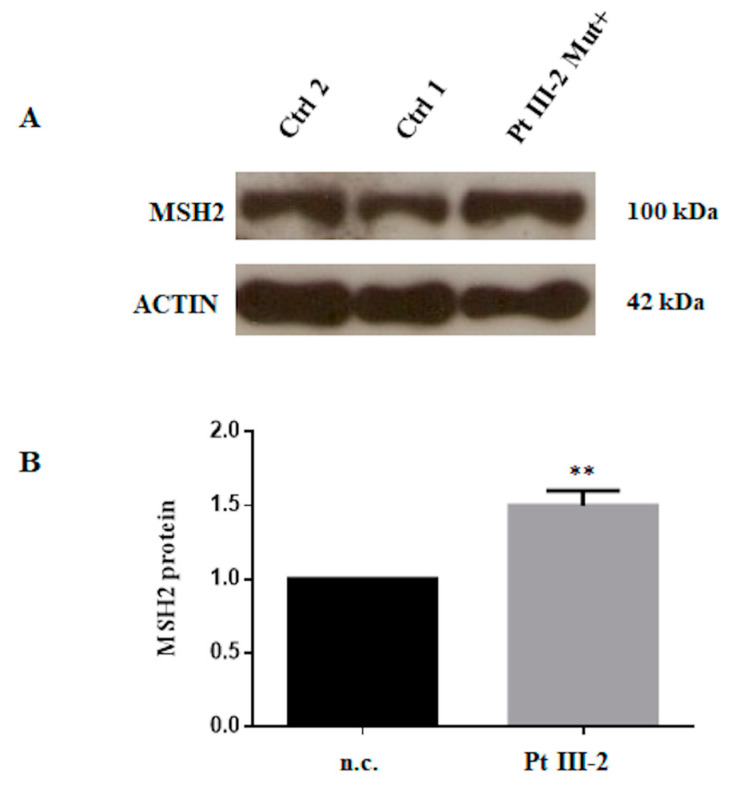
Patient protein analysis. (**A**) Western blot assay of MSH2 performed on protein extracts from peripheral blood cells of index-patient III-2 compared to two normal controls. Actin was used as internal positive reference. (**B**) Histogram showing density of MSH2 protein normalized versus actin protein and compared to the mean of two negative controls, controls 1 and 2 (n.c.). Density of the electrophoretic bands was obtained with ImageJ software. Data represent three independent experiments (mean ± SD). ** Statistical significance was determined by one-way ANOVA (*p*-values were < 0.05).

To explore the possibility of *MSH2* overexpression, we performed an expression assay by RT-qPCR analysis on mRNA extracted from the peripheral blood lymphocytes of these three patients. This analysis showed an increased level of *MSH2* mRNA expression in all three patients with the variant c.*226A > G, as shown in Figure 3. Then, subsequently we performed the Western blot analysis of proteins isolated from the lymphocytes of one (III-2 index-case) of these three patients. This analysis showed an increased level of MSH2 in our patient compared with the negative control, in accordance with the real-time data, as shown in Figure 4. 

### 3.4. Functional Effect of the MSH2 3′ UTR Variant on Reporter Luciferase Expression

**Figure 5:**
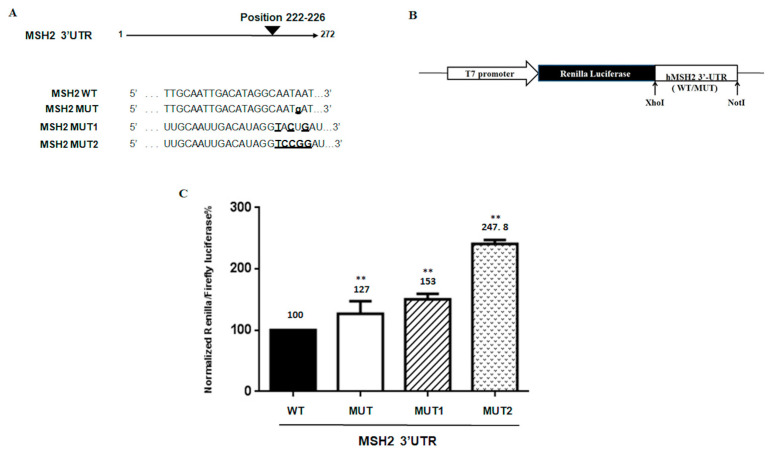
Luciferase *MSH2* 3’UTR constructs and reporter assay. (**A**) Sequences of wild type and mutant in the 3’UTR of *MSH2* of reporter constructs are shown. The mutated bases are underlined. (**B**) Schematic diagram of the luciferase reporter gene constructs. The constructs were cloned into the PSICHECK2 vector as indicated in Materials and Methods. (**C**) Luciferase activities of reporters carrying the wild type (WT) and mutated (MUT, MUT1, MUT2) MSH2 3’UTR were measured in SW480 cells. The data were normalized to the Firefly luciferase activity. Values are expressed in percentage as the mean ±SD of five determinations for samples assayed in three independent experiments. ** Statistical significance was determined by one-way ANOVA (*p*-value < 0.005).

To confirm whether the variant c.*226A > G altered the expression of upstream coding sequences, the wild type (WT) and 3′ UTR variant were cloned downstream of the *Renilla* luciferase reporter gene, as shown in Figure 5A,B. To further investigate the correlation between *MSH2* expression and the variant region, additional substitutions in this target site were generated as reported in materials and methods and shown in Figure 5A. The reporter gene constructs were transfected into SW480 cells, and the cells were collected for luciferase assay and quantitative mRNA analysis 48 h later. The results showed that the construct bearing the 3′ UTR variant consistently induced higher luciferase activity than the construct with the WT 3′ UTR, as shown in Figure 5C, according to the patient data of *MSH2* expression. Moreover, greater luciferase expression was observed in proportion to the number of additional mutations, as shown in Figure 5C.

## 4. Discussion

The study of a heterozygous single base substitution in the *MSH2* 3′ UTR, namely c*226A > G identified in a LS family, allowed us to reach some interesting conclusions. This variant was identified in three affected patients with LS-related cancer. Point variants in other *MMR* genes (*MLH1*, *MSH6, PMS2, MLH3,* and *MSH3*) [8,9,10,20], and large rearrangements in these genes (*MLH1, MSH2, MSH6,* and *PMS2*) [11,12,21] were not identified in these patients. MSI analysis revealed instability in two dinucleotide repeats, D5S346 and D18S58 (of which only one repeat is of the Bethesda panel), and then a MSI-Low status. This apparent low instability may be attributed to selected tissue stions in which the tumor component was rather low. Therefore, the revealed instability may have been underestimated by the limits of the technique, even more if we consider that endometrial tissue is not a tumoral tissue. In addition, the MSI result is also compatible with the results of immunohistochemical analysis. In fact, IHC analysis on these same tissues revealed a normal expression of the MSH2 protein or even a likely MSH2 overexpression.

Therefore, we speculated that this likely MSH2 overexpression may be present at the germline level. To investigate this, we performed gene expression analysis on mRNA from peripheral blood by RTqPCR and by Western blotting for proteins. Both experimental procedures showed increased *MSH2* gene and protein levels. Finally, we confirmed these results by functional luciferase assays in vitro. 

For a long time, it was known that the overexpression of *MLH1* and *MSH2* leads to potentially adverse consequences: apoptosis was induced in a human cell line when these two genes were upregulated in vitro under the control of the cytomegalovirus promoter. One possible explanation is the capture, by MLH1 and MSH2, of proteins crucial for cell-cycle progression, such as proliferating the cell nuclear antigen protein, which is involved in DNA synthesis [3]. Several reports suggest that cellular levels of MMR proteins are likely to be subject to tight regulation to prevent these proteins from sequestering other factors involved in controlling the mutation rate [3,5]. Moreover, a dangerous excess of MMR proteins can also affect the homodimerization complex, as found in a study of yeast cells by Shcherbakova et al. [5], who demonstrated that a MLH1–MLH1 homodimer replaced a MLH1/PMS1/PMS2/MLH3 heterodimer, inactivating MutSα and MutSβ functions and thus resulting in a non-functional MMR complex. This concept can also be partially extended to other minor MMR genes. The overexpression of MSH3 in cultured mammalian cells selectively inactivates MutSα because MSH2 is sequestered in the MSH2–MSH3 (MutSβ) complex, resulting in the reduced MutSα-dependent repair of base–base mismatches and a strong base substitution mutator phenotype [22]. For this reason, we plan to test the effect of this variant to alter the formation of the MutSα or MutSβ heterodimers, in the near future. Finally, elevated levels of MSH2 and MSH6 have been detected in primary melanomas with poor prognosis [23,24,25]. 

Therefore, *MSH2* overexpression may determine a MMR system deficiency, with a functional loss of the MSH2 protein. 

Interestingly, this variant has also been identified in another our patients, as reported in the results stion. MSI analysis for this patient showed a MSI-high status on endometrial cancer tissue (G3). This reveals a MMR complex deficiency in this patient that could be due to this c.*226A > G variant in the 3’-UTR of the *MSH2* gene. Unfortunately, we could not confirm this result by IHC analysis. 

Many hypotheses about other causes that determine loss of function in the MMR system have been postulated, such as miRNAs that can strongly influence the repair function of the MMR complex, with consequent effects on disease progression [26,27,28,29,30,31]. Thus, this overexpression effect may be related to a loss of *MSH2* downregulation, caused by a loss of base pairing with this *MSH2* 3’-untranslated region by transcription factors or miRNA. For example, the presence of single nucleotide substitutions in miRNA target sites can create or abolish miRNA interactions with their molecular targets and, therefore, determine variations in the expression of a gene [17,32]. In silico analysis of the 3’ UTR of MSH2 reveals a putative binding site for hsa-miR-137 right in the region in which the variant c.*226A > G falls.

In this regard, the greater luciferase expression observed in our study, even in proportion to the number of additional mutations created in this region, allows us to speculate that this region, in which the variant c.226A > G* falls, may prevent binding with regulatory factors, as well as with the miRNA-137. A loss of regulation of the *MSH2* transcript would explain the observed overexpression, which does not enhance genomic stability but promotes hypermutability [33,34,35].
